# *Bacillus amyloliquefaciens* PP19 regulation of microbial communities and suppression of *Peronophythora litchii*

**DOI:** 10.1186/s40168-025-02239-y

**Published:** 2025-12-18

**Authors:** Li Zheng, Xinmin Lv, Anqi Fu, Haojie Fang, Mengbing Li, Shilian Huang, Tom Hsiang

**Affiliations:** 1 College of Agriculture and Biology, Zhongkai University of Agriculture and Engineering; Key Laboratory of Green Prevention and Control on Fruits and Vegetables in South China, Ministry of Agriculture and Rural Affairs, Guangzhou, 510225 China; 2 Institute of Fruit Tree Research, Guangdong Academy of Agricultural Sciences; Key Laboratory of South Subtropical Fruit Biology and Genetic Resource Utilization, Ministry of Agriculture and Rural Affairs; Guangdong Provincial Key Laboratory of Science and Technology Research on Fruit Tree, Guangzhou, 510640 China; 3 School of Environmental Sciences, University of Guelph, Guelph, ON N1G 2W1 Canada

**Keywords:** Litchi downy blight, *Bacillus amyloliquefaciens* PP19, Exocarp microecology, Bacteriostasis, Microbial interspecies interaction

## Abstract

**Background:**

Litchi downy blight (LDB) is a major disease affecting litchi (*Litchi chinensis*), damaging fruits, inflorescences, and leaves, and significantly hindering the development of the litchi industry in China and globally. *Bacillus amyloliquefaciens* PP19 has demonstrated significant biocontrol efficacy against LDB, but its mechanism of action remains unclear.

**Results:**

This study used microbiome analysis and bacterial interaction studies to investigate the biocontrol mechanism by which PP19 regulates core microbial communities on litchi exocarps to suppress LDB. First, 16S rRNA diversity analysis revealed that PP19 pretreatment effectively prevented bacterial diversity imbalances caused by *Peronophythora litchii* infection, maintaining microbial stability by regulating the abundance of specific genera (*Actinomycetospora*, *Paenibacillus*, and *Spirosoma*). Microbial interaction networks and functional prediction revealed that PP19 might modulate bacterial motility pathways, resulting in changes to the abundance of specific microbial communities on litchi exocarps. These changes facilitated the formation of a core microbiome negatively correlated with the abundance of *P. litchii*. By isolating and genetically identifying 83 cultivable bacterial strains from litchi exocarps and using correlation analysis, 16 candidate strains with potentially significant interactions with PP19 and *P. litchii* SC18 were identified. Plate antagonism, liquid co-culture, and leaf biocontrol efficacy analyses ultimately identified four representative strains (*Sphingomonas* sp. F14, *Rhizobium* sp. F26, *Pseudomonas* sp. F32, and *Enterobacter cloacae* F63) with significant interactions with either PP19 or *P. litchii*. Interaction, motility, and biofilm production analyses showed that PP19 interacted with the four litchi exocarp bacteria to prevent disease through various mechanisms, and enhanced their motility and biofilm production to varying degrees.

**Conclusions:**

PP19 regulates core microbial communities on litchi exocarps, maintaining community stability and enriching interacting strains which together inhibit the growth of *P. litchii*, thereby achieving biocontrol efficacy.

Video Abstract

**Graphical Abstract:**

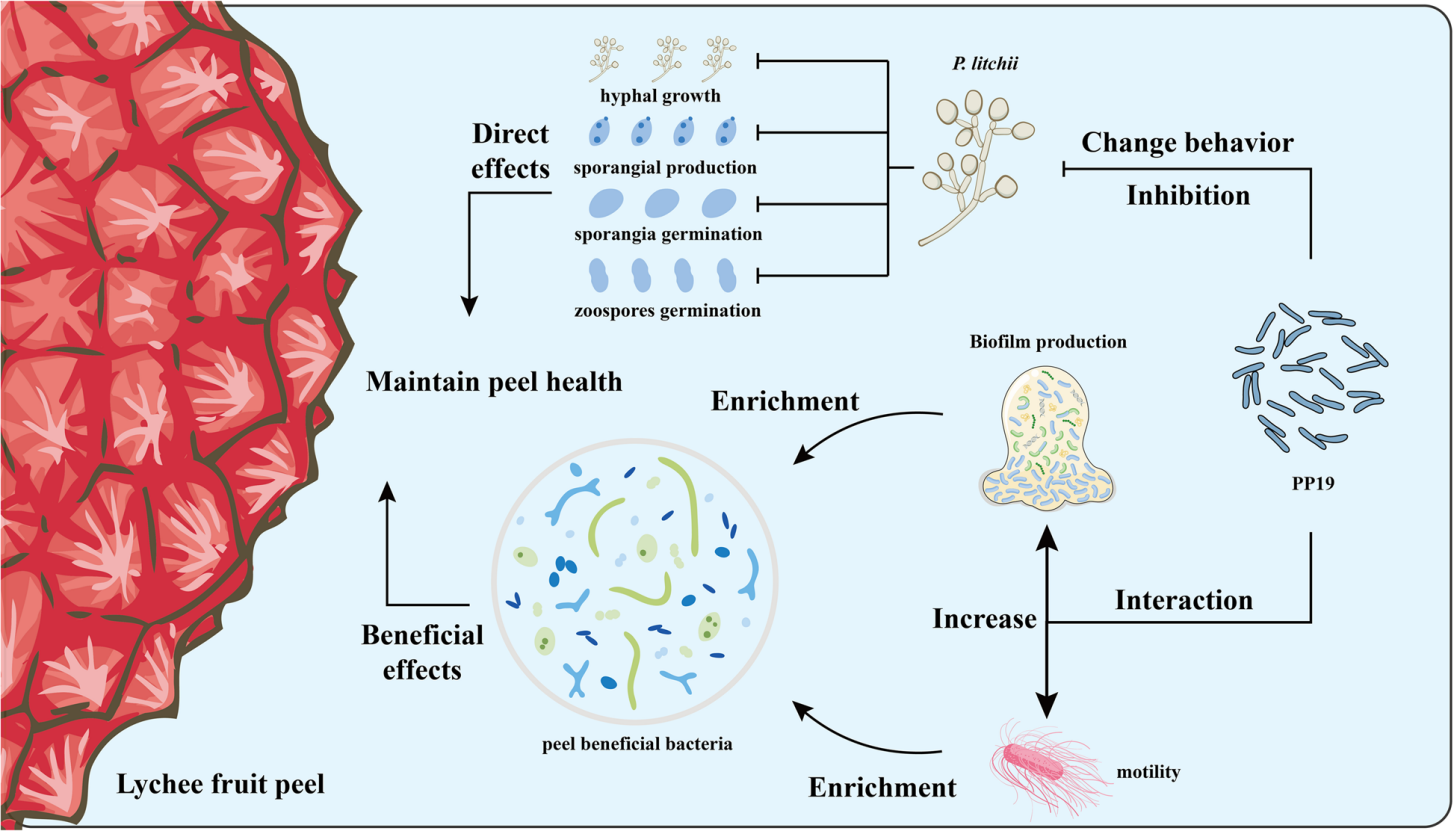

**Supplementary Information:**

The online version contains supplementary material available at 10.1186/s40168-025-02239-y.

## Introduction

Litchi (*Litchi chinensis* Sonn.) is native to China and widely cultivated in tropical and subtropical regions [1, 2]. Litchi downy blight (LDB), caused by the pathogen *P. litchii*, severely affects litchi production, often destroying 20 ~ 30% of the fruit production annually [3, 4]. Currently, chemical control, primarily using dimethomorph, is the main method for managing LDB in China. However, prolonged pesticide use can result in issues such as pesticide residues, drug resistance, and environmental pollution [5]. As a result, biological control offers a more sustainable, environmentally friendly, and potentially more effective alternative [6]. Among biocontrol agents, Bacillus species exhibit remarkable efficacy against fungal and bacterial pathogens. For instance, *Bacillus sp*. G4L1 reduces tomato wilt caused by *Ralstonia solanacearum* [7], while *Bacillus altitudinis* B1-15 inhibits *Botrytis cinerea* in tomato gray mold [8]. In rice, *Bacillus amyloliquefaciens* S170 and *Bacillus pumilus* S9 control blast by antagonizing *Magnaporthe oryzae* and colonizing plants [9], and endophytic *Bacillus* strains suppress sheath blight and bacterial panicle blight [10]. Collectively, these studies highlight the potential of *Bacillus* species for biological control of diseases in both staple and horticultural crops.

Increasing evidence shows that regulating microbial community composition and interactions is a key mechanism for biocontrol bacteria to inhibit pathogens and promote plant health [11, 12]. The plant surface microbiome, termed the "second genome," maintains plant health through its diversity and stability, occupying ecological niches to prevent pathogen invasion [13–16]. For example, stable soil microbial diversity effectively controls *Fusarium* diseases [17], while community imbalance facilitates pathogen invasion [18, 19]. Biocontrol bacteria can modify microbial communication networks to reduce disease [20]. *Pseudomonas fluorescens* pc78, for instance, alters tomato rhizosphere microbiome structure, increasing beneficial bacteria and decreasing pathogens [21]. Pseudomonas diversity also correlates with reduced Ralstonia solanacearum density and wilt incidence [22]. *Bacillus* species (e.g., *B. subtilis*, *B. thuringiensis*, *B. amyloliquefaciens*) likely enhance plant health by modulating microbial communities across diverse crops and environments [21–30].

Our preliminary research found that *Bacillus amyloliquefaciens* PP19 significantly inhibits the growth of *P. litchii*, enhances the activity of disease-resistant enzymes in litchi fruit during storage, and alters the microbial community on the exocarps [6, 30]. In this study, we first analyzed the impact of PP19 on the stability of microbial communities on the litchi exocarps, by exploring their role in microbial community stability and the maintenance of ecological balance. We then assessed the influence of PP19 on the interrelationships among microbial genera associated with the exocarps and their potential interaction pathways, elucidating pivotal roles within the microbial interaction network. Finally, we explored the interaction mechanisms between PP19 and its interacting strains, as well as synergistic effects on the structure and function of the microbial community. This study provides important theoretical support for the biological control of LDB. By investigating PP19 and its associated microbial community associated with litchi in detail, we aim to offer new perspectives and methodologies for future biological control strategies.

## Materials and methods

### Strains and materials

*Bacillus amyloliquefaciens* PP19 was isolated from the surface of litchi exocarps. The strain *P. litchii* SC18 was provided by Professor Zide Jiang from the Fungal Laboratory, Department of Plant Pathology, South China Agricultural University, who isolated it from a typical occurrence of LDB. Litchi fruits of the variety 'Feizixiao' were obtained from the Longan Germplasm Resource Orchard in Guangzhou, Guangdong Province. Litchi leaves of the variety 'Dongguancuirou' were obtained from the National Litchi Germplasm Resource Orchard in Guangzhou.

The preparation of PP19 starter inoculum, fermentation broth, clear supernatant, and bacterial suspension, as well as the spore suspension and swarm spore suspension of SC18, followed the methods described by Zheng [4].

### Detection of *P. litchii* content in litchi exocarps

Total RNA was extracted from the litchi exocarps inoculated with* P. litchii* using the RNAprep Pure Polysaccharide Polyphenol Plant Total RNA Extraction Kit (DP441). cDNA was synthesized using the PC44-THERMOscript 1 st Strand cDNA Synthesis Kit (PC4402) to synthesize the first strand of cDNA. Primers for quantitative internal reference actin of *P. litchii* (*P. litchii*-ACTqRT-F: ACATTGCCCTGGACTTCG and *P. litchii*-ACTqRT-R: AGCTCCTTGGTCATACGC) and internal reference primers for litchi (Litchi-ACTqRT-F: CGGGAAATTGTCCGTGAC and Litchi-ACTqRT-R: GAGGACTTCTGGGCAACG) were designed online using Primer 3 software. qRT-PCR analysis was performed using the QuantStudio 3 Real-Time PCR System. The reaction system was conducted according to the instructions of the PC60-2 × SYBR Green qPCR Mix (Low ROX) kit. Each sample was repeated three times, and the relative gene expression was calculated using the 2^−ΔΔ^CT method [31].

### 16S rRNA sequencing and analysis of litchi exocarp microbiota

A bacterial culture suspension of PP19 (5 × 10⁷ CFU/mL) or sterile water was sprayed on attached'Feizixiao'litchi fruits (5 L per treatment, 60–100 fruits) in the Longan Germplasm Resource Orchard until run-off, with four replicate branches per treatment. The bacterial culture suspension was applied three times before harvest (18, 8, and 3 days pre-harvest). On the day of harvest, fruits were harvested and transported to the laboratory, where 400 mL of sterile water was sprayed on each treatment group (four replicates), and the fruit were placed into storage boxes (dimensions: 323 × 220 × 100 mm), and the boxes sealed. After 24 h, the fruits were inoculated with a sporangial suspension spray of SC18 (5 × 104 sporangia/mL) at 10 mL per box. The treatments were as follows: TDS (PP19 + SC18), CDS (sterile water + SC18), TRS (PP19), and CRS (sterile water). Samples were collected at 0 h post-treatment (hpt with last application of PP19), 0 h post-inoculation (hpi with SC18), and at 60 and 72 hpi. Sterile scalpels were used to collect litchi exocarps, which were then cut into 1 × 1 cm pieces using sterile scissors. Each sample consisted of four biological replicates, with each biological replicate prepared using the exocarps of three litchi fruits. The collected exocarp samples were rapidly cooled in liquid nitrogen and subsequently stored at −80℃.

Litchi exocarp samples (5 g per tube) were placed into centrifuge tubes containing 50 mL of sterile physiological saline and shaken at 200 rpm for 30 min to dislodge microorganisms attached to the exocarps. The suspensions were then gently vortexed for 30 s and were filtered through 0.45 µm membranes and centrifuged at 8,000 × g for 10 min to collect microbial pellets. The total DNA of microorganisms from exocarps were extracted using a PureLink™ Microbiome DNA Purification Kit (Thermo Fisher Scientific, A29790). The V3-V4 region of the 16S rRNA gene was amplified by PCR of extracted DNA with primers F341 (5'-CCTAYGGGRBGCASCAG-3') and R806 (5'-GGACTACNNGGGTATCTAAT-3'). The purity and concentration of the PCR products were analyzed with a Bioanalyzer (Agilent 2100). The DNA library was prepared using the NEXTFLEX Rapid DNA-Seq Kit (Bluescape, NOVA-5188–01), and sequenced on the Illumina HiSeq 2500 high-throughput sequencing platform by Shanghai LingEn Biotechnology Co., Ltd.

Raw sequencing data were processed as follows: quality control was first performed using Trimmomatic (v0.39) [32] to trim adapter sequences and remove low-quality reads (Phred score < 20, sliding window size: 4 bp). Paired-end reads were merged using FLASH (v1.2.11) [33] with a minimum overlap of 10 bp and maximum mismatch density of 0.25. Chimeric sequences were detected and removed using UCHIME (https://drive5.com/uchime/uchime_download.html) in de novo mode. High-quality, non-chimeric sequences were clustered into operational taxonomic units (OTUs) at 97% similarity threshold using USEARCH (v9.2.64) [34], followed by singleton removal (OTUs represented by ≤ 1 read across all samples) via QIIME2 (v2021.4; https://qiime2.org/). Taxonomic annotation was assigned to OTUs using the SILVA database (v138; https://www.arb-silva.de/) with a confidence cutoff of 0.8.

### Microbial diversity analysis

Alpha diversity indices, including the observed species richness and Shannon indices for diversity, were calculated using QIIME2 and visualized using R software v3.6.3 (https://www.r-project.org/). One-way ANOVA followed by Tukey’ s least significant difference (LSD) test were performed in R software v3.6.3 to compare statistically significant differences among the different groups with at P ≤ 0.05. Dissimilarities in taxonomic diversity among different samples were visualized using beta-diversity of principal coordinates analysis (PCoA) with a Bray–Curtis distance matrix of taxon relative abundances using the VEGAN v 2.5–7 package in R [35]. Potential microbial indicators among the different groups were predicted using the Labdsv v2.0–1 package (https://CRAN.R-project.org/package=labdsv) in R.

### Microbial differences and interaction analyses

Linear discriminant analysis (LDA) of effect size (LEfSe) was used to characterize the biomarker features in each group according to LDA scores (log10) > 3 and *P* < 0.05 [36]. The Kyoto Encyclopedia of Genes and Genomes (KEGG, https://www.genome.jp/kegg/) pathway analysis of the OTUs was performed using Tax4Fun (https://github.com/bwemheu/Tax4Fun2). The pairwise associations of PP19 and other microbiota were estimated using the Spearman correlation coefficient based on the relative abundance in each sample. Connection retention was saved while satisfying the thresholds of absolute correlation value > 0.7 and *P*-value < 0.05, and the cutoff for the selected genus was set to presence in more than half of the samples [37]. Microbiome interaction networks were visualized using Cytoscape v3.8.2 (https://cytoscape.org/). Finally, the correlation between relative abundance of microbiota and the abundance of microbial functional categories in samples were estimated using the Spearman correlation coefficient comparing the relative abundance of each microbial taxon to the abundance of each functional category across all samples.

### Isolation and cultivation of bacteria from litchi exocarps

Bacterial isolation was conducted concurrently with the collection of exocarp samples for sequencing at 0 hpt with PP19, and 0, 60, 72, and 84 hpi with SC18. At each sampling time for each treatment, 3 g litchi exocarps were homogenized with 27 mL of sterile 0.85% NaCl and sterile glass beads, followed by shaking at 180 rpm for 30 min, and standing for 5 min at room temperature. Each suspension was filtered through sterile gauze, and diluted to obtain 10⁻1 bacterial spore suspensions. A 0.1 mL aliquot of each suspension was mixed with 900 μL of sterile 0.85% NaCl to prepare a 10⁻2 dilution of bacterial spores, which was serially diluted to 10⁻⁶. A 100 μL aliquot of each of the 10⁻4, 10⁻5, and 10⁻⁶ dilutions was plated on Reasoner's 2 A agar (R_2_A) solid medium [38], with three replicates per gradient, and incubated upside down at 30℃ for 48–72 h. Distinct single colonies exhibiting unique morphological and growth characteristics were selected and further purified on LB (Luria–Bertani) agar plates. During the purification process, colonies were evaluated for consistent morphology, color, growth rate, and odor. Once all colonies on the plate displayed uniform characteristics, a single isolated colony was chosen for preservation in sterilized glycerol, and stored at −80℃.

### Genetic diversity analysis of litchi exocarp bacteria

All bacterial strains stored at −80℃ were streaked onto LB agar, and incubated upside down at 30℃ for 48–72 h for revival. Single isolated colonies were placed into 5.0 mL of LB broth, and cultured at 30℃ with shaking at 200 r/min for 18–20 h to produce bacterial fermentation broth. A 1.5 mL aliquot of the fermentation broth for each isolate was collected, and bacterial DNA was extracted using the Tianamp Bacteria DNA Kit (Tiangen Biotech Co., Ltd., Beijing, China). PCR amplification of bacterial 16S rRNA sequences was performed using Taq PCR Master Mix (Tiangen Biotech Co., Ltd., Beijing, China), with bacterial DNA as the template. The primers used were U8-27 (F): 5'-AGAGTTTGATC(AC)TGGCTCAG-3'and L1494-1514 (R): 5'-CTACGG(AG)TACCTTGTTACGAC-3'. The PCR products were sent to Sangon Biotech Co., Ltd. (Shanghai, China) for Sanger sequencing. Multiple 16S rRNA sequences were aligned using ClustalW in MEGA software version 11 (https://www.megasoftware.net/). A dendrogram was constructed from the aligned sequences using the Neighbor-Joining algorithm with 1000 bootstrap replicates.

BOX-PCR fingerprinting analysis was performed using total bacterial DNA as a template. BOX-PCR reactions were done with primer BOXAIR: 5’-CTACGGCAAGGCGACGCTGACTGACG-3’. The BOX-PCR gel electrophoresis profiles were subjected to cluster analysis. The lanes corresponding to different strains contained multiple DNA bands, and DNA bands with the same migration rate were considered to have similar DNA sequences. If a DNA band were present at a band position, it was marked as "1"; otherwise, it was marked as "0". The marked data were analyzed using the UPGMA method in NTSYS software (https://www.appliedbiostat.exetersoftware.com/ntsyspc/ntsyspc.html) to perform cluster analysis, and a dendrogram was constructed using a 80% similarity threshold as the standard. Bootstrap resampling (1,000 iterations) was applied to assess the robustness of cluster nodes, with values ≥ 70% indicating significant phylogenetic grouping.

### Inhibition by biocontrol bacteria of *P. litchii* mycelial growth

Assessment of inhibition of *P. litchii* mycelial growth followed Zheng [4], with the following modifications. A mycelial block of SC18 was placed at the center of a CJA-PDA plate. After that, 10 μL of the test bacterial fermentation broth (OD_600_ = 1) from multiple bacterial agents were placed at four equidistant points around the pathogen. The inhibition rate was calculated as follows: ((Diameter of pathogen in control group—Diameter of pathogen in treatment group)/Diameter of pathogen in control group) × 100%.

### Inhibition of sporangial production of *P. litchii* by biocontrol bacteria

A agar punch was used to extract a fresh 5-mm hyphal plug from the outer margins of a colony of of *P. litchii* stored grown on CJA agar. The plug was placed onto CJA medium and incubated at 28℃ for 7 days until the pathogen fully covered the medium. Eight mycelial plugs (5 mm in diameter) were taken from the colony edge and transferred to fresh CJA-PDA plate. Aliquots of 10 mL of biocontrol bacterial fermentation broth (OD_600_ = 1), bacterial suspension (OD_600_ = 1), or supernatant (passed through a 0.22 μm sterile filter) was added to the Petri dishes to submerge the *P. litchii* mycelial plugs. The liquid was replaced every 40 min, repeated 4–5 times, to induce sporangial production. Sporangial production was assessed and photographed under a microscope. In the control group, sterile water was used instead of the bacterial solutions. Inhibition level was calculated as follows: (sporangial production in the control group—sporangial production in the bacterial treatment group)/sporangial production in the control group × 100%.

### Inhibition of *P. litchii* sporangial and zoospore germination of by biocontrol bacteria

We followed the experimental methods of Xu [39] and Kao [40]. Preparation of the spore suspensions and swarm spore suspensions of *P. litchii* SC18 followed the methods described by Zheng [4]. To prepare the experimental treatments, 4.5 mL of the sporangial suspension (5 × 104 sporangia/mL) was mixed with 0.5 mL of biocontrol bacterial fermentation broth (OD_600_ = 1), bacterial suspension (OD_600_ = 1), or supernatant (filtered through a 0.22 μm sterile filter) in 2 mL tubes for each tested bacterium. To prepare the control, 4.5 mL of sporangial suspension was mixed with 0.5 mL of sterile water in 2 mL tubes. The tubes were incubated at 30℃ with shaking at 180 r/min. Each treatment included six replicates. At 6 h and 9 h, 1 μL suspension was taken from each tube, and the germinated and ungerminated sporangia were observed and counted under a microscope. For each replicate, 200 sporangia were examined. The determination of zoospore germination level and inhibition level was performed by replacing the sporangium suspension in the above method with a zoospore suspension. The germination level was assessed as follows: (number of germinated spores/total number of spores) × 100. The inhibition level was calculated as follows:

$$\left[\left(\mathrm{germination}\;\mathrm{level}\;\mathrm{of}\;\mathrm{control}\;\mathrm{group}\;-\;\mathrm{germination}\;\mathrm{level}\;\mathrm{of}\;\mathrm{treatment}\;\mathrm{group}\right)/\mathrm{germination}\;\mathrm{level}\;\mathrm{of}\;\mathrm{control}\;\mathrm{group}\right]\;\times\;100.$$

### Analysis of interaction types among bacteria from litchi exocarps

Following the method described by Li [41], PP19 and 16 bacterial strains were paired in combinations to determine their level of interaction. For BCA (Biological Control Agents) Experimental Groups, 800 μL of NA medium and 100 μL of sterile water were added to each 2 mL tubes, followed by 100 μL of a single biocontrol bacterial culture suspension (OD_600_ = 1) or a combination (individual strains mixed at OD_600_ = 1 in a 1:1 volume ratio). Six replicates were used for each treatment. For BCAs plus SC18 Experimental Groups, the method was the same as above, except that sterile water was replaced with 100 μL of SC18 zoospore suspension. For both experimental groups, the cultures were incubated at 28℃ and 200 rpm for 48 h, and OD_600_ of the bacterial culture suspensions was measured. The mean interaction factor (MIF) was calculated as follows: MIF = log(CPi + j/MPi + MPj) where CPi + j represents the OD_600_ of the combined bacterial agent after 48 h of culture, and MPi and MPj were the average OD_600_ values of the two individual strains cultured separately for 48 h. When MIF > 0, the interaction was classified as positive; when MIF < 0, the interaction was classified as negative.

### Analysis of the biocontrol effect of bacteria

Healthy litchi leaves and fruits were treated as follows: test bacterial culture suspensions diluted 50-fold (1 × 10⁷ CFU/mL) were sprayed onto the leaves and fruits, while the control group was sprayed with sterile water. Each treatment included three replicates, with three detached branches with fruit per replicate. The branches and fruits were placed in storage boxes with sterile filter paper at the bottom. At 24 h after bacterial treatment, a sporangial suspension of the PP19 pathogen (5 × 104 sporangia/mL) was applied. At 36 hpi, the disease index was calculated following Zheng [4].

### Analysis of interactions between bacteria

Attraction between colonies of biocontrol bacterium PP19 with litchi exocarp bacteria was evaluated following the methods of Berendsen [18]. PP19 and the four exocarp bacterial strains, F14, F26, F32, and F63 were each placed into 5 mL of King’s medium B and incubated overnight at 28℃ with shaking at 180 rpm. The bacterial cultures were standardized to OD600 = 0.1. The square Petri dishes (10 cm by 10 cm) containing the King’s medium B were visually divided into left and right sections.Seven 1 μL suspension droplets of each test strain were placed along one side of the plate at different distances from the center line. Seven 1 μL suspension droplets of another test strain were placed along the other side of the plate to form a V pattern so that the distances between the colonies differed. This setup allowed for the observation of bacterial interactions as colonies expanded toward each other. The plates were sealed with Parafilm and incubated at 28℃ for 15 days, after which colony diameters were measured to assess the interaction effects.

The chemotaxis of four strains (F14, F26, F32, and F63) toward the supernatant of PP19 was evaluated using a capillary assay. The chemoattractant was a PP19 bacterial suspension at OD_600_ = 1, prepared in 10 mM Tris–HCl buffer (pH 7.0). The control chemoattractant consisted of 10 mM Tris–HCl buffer (pH 7.0). A 5 μL capillary tube (WHEATON, 851,321) was filled with 5 μL of the chemoattractant, and one end was sealed with molten paraffin wax. The test bacteria were suspended in 10 mM Tris–HCl buffer (pH = 7.0) and adjusted to an OD_600_ of 1.0. The capillary tube was inserted into a 5 mL test tube (CITOGLAS, 84,004–0110) containing 400 μL of the bacterial suspension. The test tubes were incubated vertically for 90 min at 28℃. The capillary tubes were then removed and washed externally with 500 μL 10 mM Tris–HCl buffer (pH = 7.0). The sealed end was broken, and the contents were expelled into a 2 mL centrifuge tube. After adding 1 mL 10 mM Tris–HCl buffer, the sample was diluted 100-fold, and 100 μL was spread on LB plates. Plates were incubated in the dark at 28℃ for 40 h. CFUs were evaluated and photographed. Each test was repeated four times. The chemotactic response was assessed based on the number of bacteria in the capillary tube, using the formula: CFU = log (dilution factor) + log (CFU count).

For the analysis of swarming motility, a single colony of active PP19 and four litchi exocarp bacterial strains (F14, F26, F32, and F63) were placed into 5 mL of LB broth, and cultured at 28℃ with shaking at 200 rpm for 18–20 h. The bacterial cultures were resuspended in sterile water and adjusted to an OD_600_ = 1.0. The experimental groups consisted of mixed bacterial suspensions of PP19 and the four litchi exocarp strains at different ratios (1:1, 1:2, 2:1). In the control group, sterile water replaced the bacterial suspensions of the four litchi exocarp strains. A 3 μL aliquot of the mixed bacterial suspension was spotted at the center of a Petri dish containing solid LB medium and incubated at 28℃ for 18 h. The swarming range of the bacteria was then observed and measured, with each assay repeated six times.

For biofilm production analysis, a mixture of 100 μL of culture medium and 1 μL of test bacterial culture suspension were added to a 96-well cell culture plate, with each bacterial combination repeated 12 times. After incubation at 28℃ and 150 rpm for 18 h, the culture medium was discarded. Then, 150 μL of 0.1% (w/v) crystal violet was added to each well and stained at room temperature for 20 min, followed by removal of the dye solution. Wells were washed three times with 200 μL of sterile water, dried, and then 200 μL of 95% ethanol was added to each well and left to stand for 15 min. The absorbance at 595 nm was measured to quantify biofilm production.

### Data analyses

Statistical analyses were performed using SPSS 25.0 (https://www.ibm.com/spss). Significant differences among more than two treatments were analyzed using one-way ANOVA and Duncan's multiple range test; differences between two treatments were analyzed using a t-test. All tests used significance set at P < 0.05. Graphs were created using GraphPad Prism 8.0 (https://www.graphpad.com/).

## Results

### PP19 regulates core microbial communities to mitigate *P. litchii* infection in litchi fruits

**Fig. 1:**
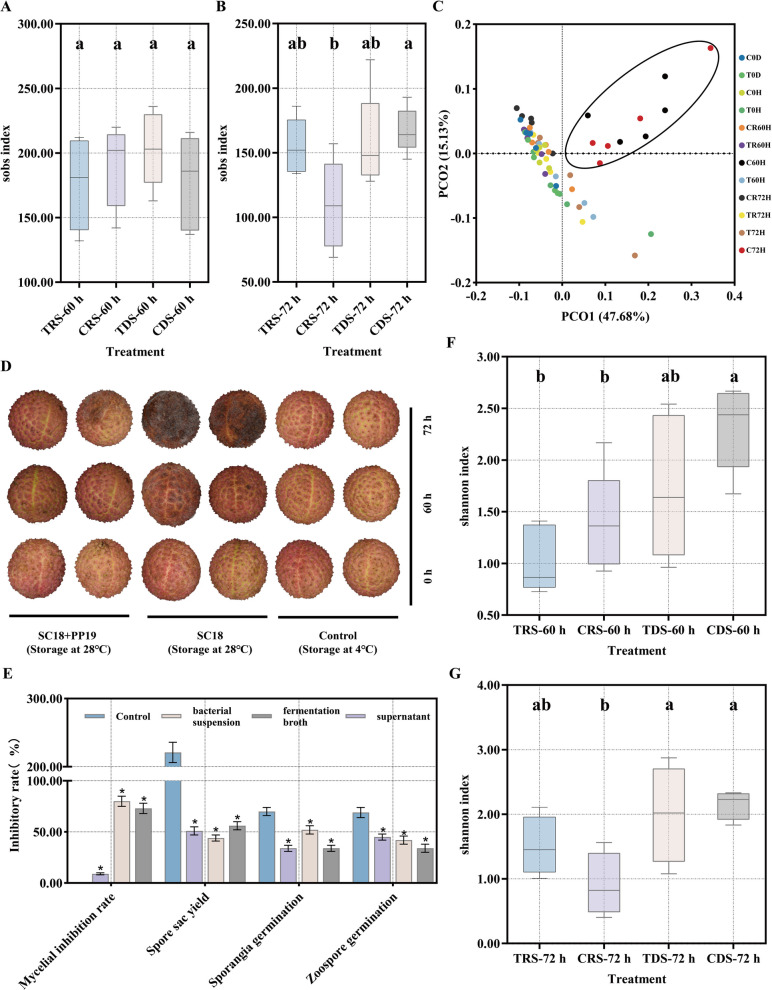
Changes in bacterial community diversity and the inhibitory effects of PP19 under different treatments. **A**, **B** α-diversity represented by the SOBS index. **F**, **G** α-diversity represented by the Shannon index. **C** β-diversity analysis represented by principal coordinate analysis (PCoA) of bacterial communities on the surface of litchi peels under different treatments. **D** Phenotypic changes in litchi fruit infected by *Peronophythora litchii* under different treatments. **E** Inhibitory effects of PP19 on *P. litchii* mycelial growth, sporangia production, sporangial germination, and zoospore germination

PP19 maintained the stability of bacterial communities on litchi exocarps following SC18 infection. An analysis of bacterial diversity on litchi exocarps under different treatments revealed no significant differences among the four groups at 60 h post-treatment (Fig. 1A): CDS (without PP19, with SC18), CRS (without PP19, without SC18), TDS (with PP19, with SC18), and TRS (with PP19, without SC18). However, at 72 h post-treatment, bacterial diversity in the CRS group was significantly lower than in the CDS group (Fig. 1B). β-diversity analysis using PCoA showed that the CDS group at 72 h was distinct from the other groups (Fig. 1C), indicating that *P*. *litchii* SC18 presence significantly altered bacterial diversity on litchi exocarps.We detected the relative abundance of SC18 on the surface of litchi exocarps and calculated the relative abundance of PP19 based on the 16S rRNA sequencing results. The results showed that the relative abundances of PP19 and SC18 on litchi exocarps were negatively correlated (Supplementary Fig. S1). PP19 reduced the damage caused by SC18 to postharvest litchi fruits and significantly inhibited hyphal growth, sporangial production, and the germination of both sporangia and zoospores of SC19 (Fig. 1D, E). After PP19 treatment (72 hpt), no significant differences in α-diversity or Shannon index values were observed between the TDS, TRS, and CRS groups (Fig. 1F, G). This suggested that PP19 pretreatment effectively alleviated bacterial community imbalances caused by *P*. *litchii* infection.

**Fig. 2:**
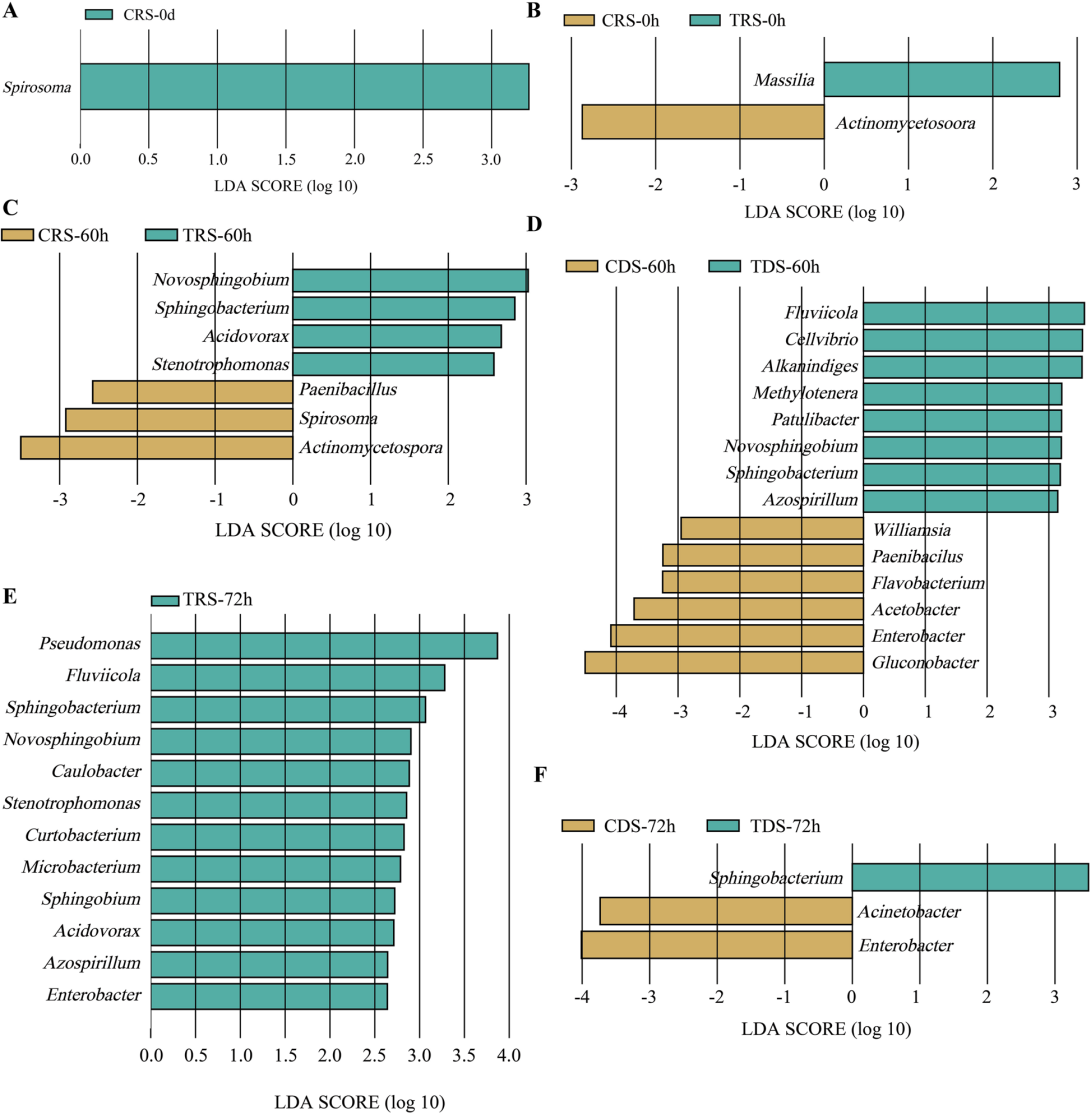
Significantly altered bacterial genera in the litchi peel surface bacterial community under different treatments, represented by linear discriminant analysis (LDA) scores (log10 > 3, *P* < 0.05)

BLASTn and LEfSe analyses revealed significantly altered bacterial communities on litchi exocarps under different treatments, and only the abundance of the genus *Spirosoma* showed significant differences between TRS-0d and CRS-0d samples which were right before harvest (Fig. 2A). The abundance of *Massilia* significantly increased in TRS-0h, while *Actinomycetospora* significantly decreased (Fig. 2B). At 60 and 72 h hpt, the abundance of *Novosphingobium*, *Sphingobacterium*, *Acidovorax*, and *Stenotrophomonas* significantly increased in the TRS group (Fig. 2C, D). These results indicated that PP19 treatment can alter the abundance of specific bacterial species on litchi exocarps. In contrast, at 60 and 72 h post-treatment, the abundance of *Sphingobacterium* in the TDS group was significantly higher than in the untreated group (CDS), whereas *Enterobacter* was significantly lower (Fig. 2E, F). The genera *Actinomycetospora*, *Paenibacillus*, and *Spirosoma*, which showed significant changes, have been extensively studied as biocontrol agents [41, 42], and they became dominant on litchi exocarps after PP19 treatment. We propose that *P. litchii* can cause microbial community imbalance on litchi exocarps by altering the abundance of specific bacterial species, ultimately leading to LBD (Litchi downy blight) development, and that PP19 can maintain microbial balance on litchi exocarps by regulating specific bacterial species, thereby mitigating *P. litchii* infection.

**Fig. 3:**
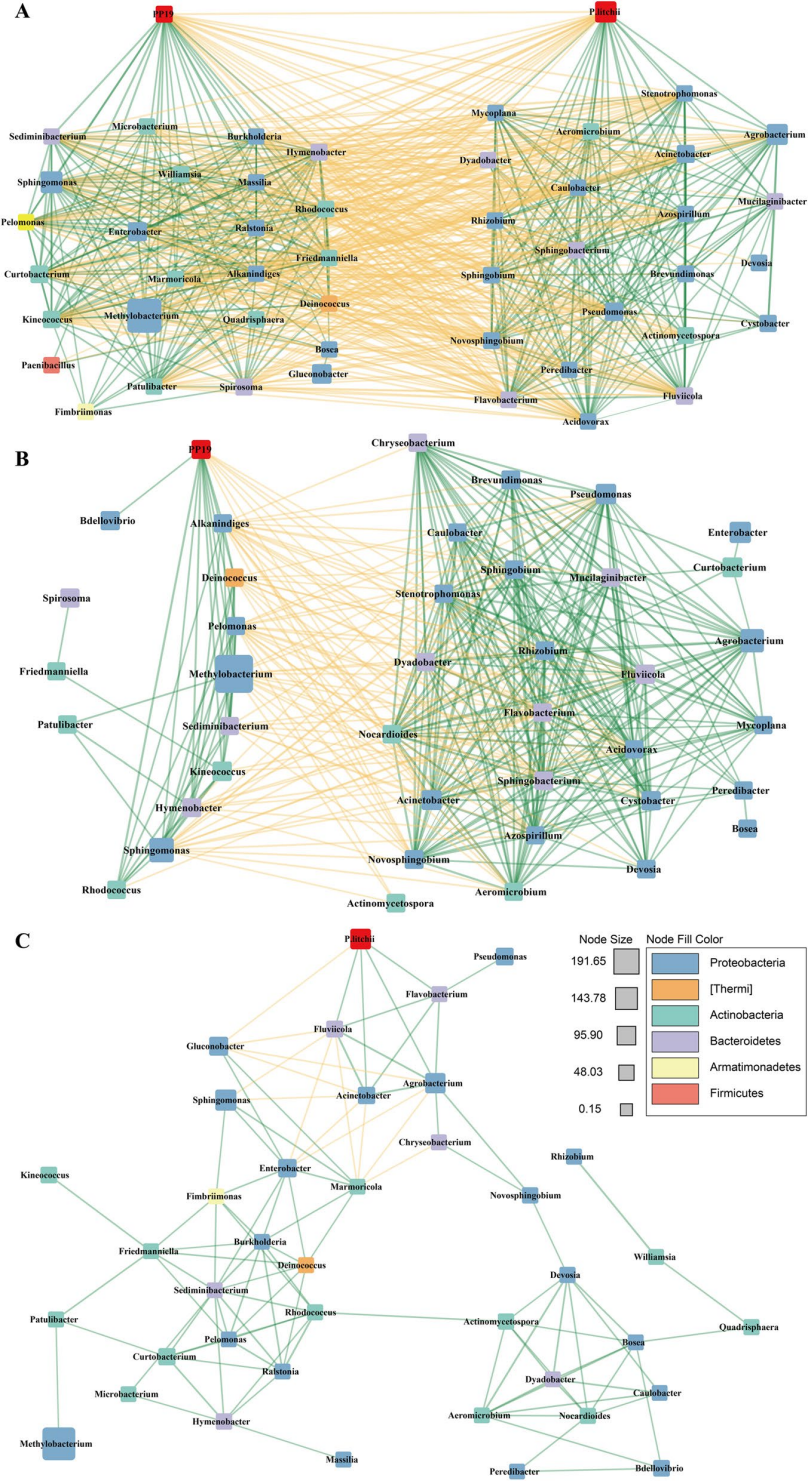
Interaction relationships between different bacterial genera and PP19 or SC18 in the litchi peel bacterial community under TDS (**A**), TRS (**B**), and CDS (**C**) treatments. Interaction networks are based on an absolute correlation value > 0.7 (*P* < 0.05), with selected genera present in more than half of the samples as the cutoff criterion. Green lines indicate a collinear relationship between two connected nodes, while yellow lines indicate a mutually exclusive relationship. Different colors of nodes are used to distinguish different Phyla, and the size of nodes represents the correlation value

We calculated the Spearman correlation coefficients between the abundance of PP19, SC18, differentially abundant genera, and other core genera (detected in > 50% of samples). In the TRS group, interactions among PP19-associated genera were divided into two modules: genera within each module were positively correlated, while genera between modules were negatively correlated (Fig. 3A). In the CDS group, only one genus (*Gluconobacter*) was directly negatively correlated with SC18 abundance, significantly less than those positively correlated with SC18 (Fig. 3B). Notably, except for *Rhodococcus*, all genera indirectly negatively correlated with SC18 were mutually exclusive with those positively correlated among the different treatments. In the TDS group, genera on litchi exocarps formed two modules that were significantly positively correlated with PP19 and SC18, respectively, and were mutually exclusive (Fig. 3C). Compared to the CDS group, the number of genera negatively correlated with SC18 increased significantly under TDS treatment. These findings suggested that PP19 influenced microbial abundance on litchi exocarps, forming a core microbiota directly negatively correlated with *P. litchii* and inhibiting its growth.

**Fig. 4:**
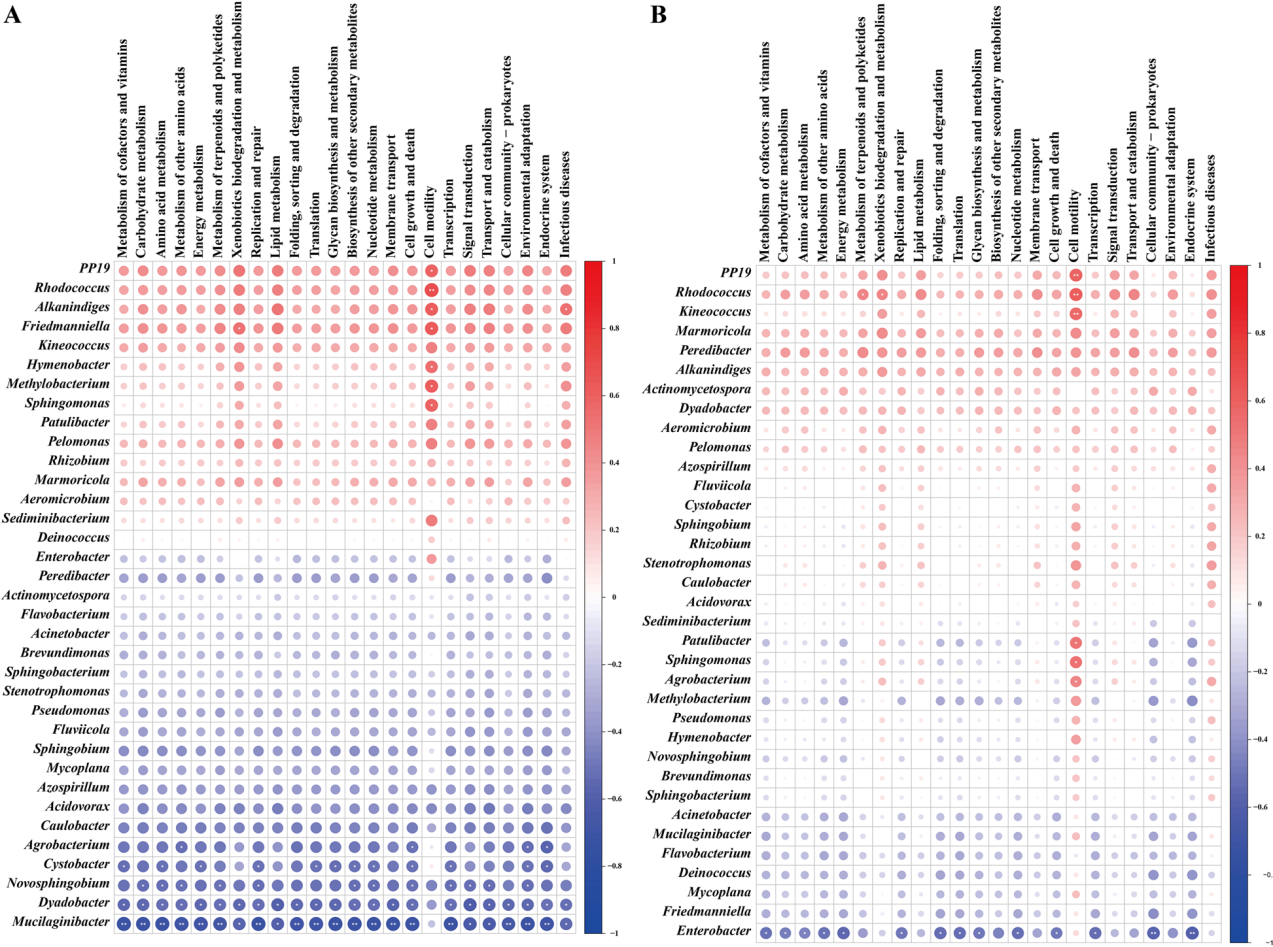
Functions of PP19 and its core bacterial community in the litchi peel microbiome under TDS (**A**) and TRS (**B**) treatments, characterized using Spearman correlation coefficients. Red and blue color blocks indicate positive and negative correlations, respectively. The size of the color blocks represents the magnitude of the correlation value, and the asterisks (*) in the color blocks denote whether the correlation is statistically significant

The functional properties of PP19 and hub microbiota were predicted based on the Spearman correlation coefficient between bacterial abundance and the abundance of pathways within total microbiome. Accordingly, in the TDS group, PP19 significantly affected the cell motility pathway (Fig. 4A). The genera *Rhodococcus*, *Methylobacterium*, and *Sphingomonas*, which were co-occurrent with PP19, were also associated with the cell motility pathway. Conversely, bacterial genera antagonistic to PP19, such as *Mucilaginibacter*, *Dyadobacter*, *Cystobacter*, and *Novosphingobium*, showed no association with most functional pathways linked to PP19 (Fig. 4A). These findings suggested that genera antagonistic to PP19 may perform functions opposite to those of PP19 in the overall community. In the TRS group, PP19 and its potential symbiotic genus *Rhodococcus* correlated with cell motility pathways (Fig. 4B). However, other antagonistic genera had a weaker impact on community functions. Therefore, PP19 may inhibit the growth of *P. litchii* and its positively correlated bacteria by influencing their cell motility and interacting with specific bacterial communities on litchi exocarps.

### Isolation, cultivation, and functional validation of bacteria from litchi exocarps

**Fig. 5:**
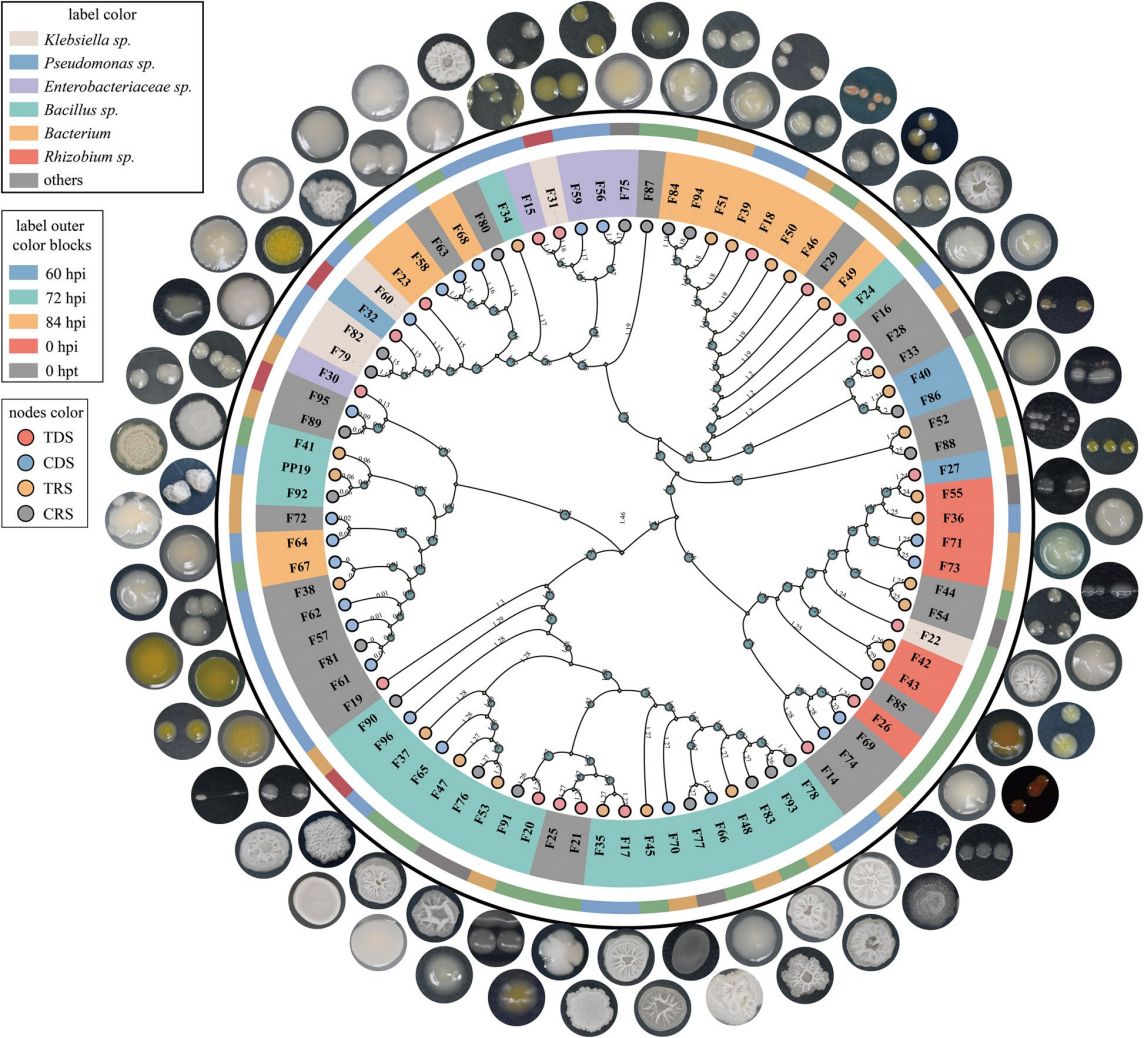
Phylogenetic analysis of 16S rRNA sequences of 83 cultivable bacterial strains isolated from litchi exocarps. The colors of different nodes in the phylogenetic tree indicate the strains isolated from different treatments: pink for the TDS treatment group, blue for CDS, yellow for TRS, and gray for CRS. The outermost color blocks corresponding to each label represent the treatment times when the strains were isolated: blue for 60 hpi (hours post inoculation), green for 72 hpi, yellow for 84 hpi, red for 0 hpi, and gray for 0 hpt (hours post treatment). The colors of different labels denote strain taxonomy: pink for *Klebsiella sp*.; blue for *Pseudomonas sp*.; purple for *Enterobacteriaceae sp*.; green for *Bacillus sp*.; yellow for *Bacterium*; red for *Rhizobium sp*.; and gray for other genera

Genetic diversity analysis of cultivable litchi exocarps bacteria. We isolated and cultured litchi exocarps bacteria from different treatment groups, obtaining 83 cultivable bacterial strains. Phylogenetic analysis based on 16S rRNA sequences classified these strains into 20 genera, with *Bacillus* being the most abundant (27.7%) and representing the dominant genus on litchi exocarps (Fig. 5). Genetic diversity analysis using BOX-PCR divided the 83 strains into 35 clusters. The two largest clusters included strains F34, F24, F17, F20, F35, and F47; and strains F65, F92, F76, F37, F78, F83, F93, F48, F45, F77, F96, and F53 (Supplementary Fig. S2). The number of cultivable bacteria varied among treatment groups, with the TDS group having the fewest strains (19) and the TRS group having the most (23) (Supplementary Table S1). Bacterial diversity also differed by treatments, with TDS showing the highest species diversity and TRS showing the lowest. This pattern was consistent with the diversity results indicated by the Shannon and Simpson indices in the 16S rRNA sequencing analysis (TDS 60 h vs. TRS 60 h; TDS 72 h vs. TRS 72 h).

**Fig. 6:**
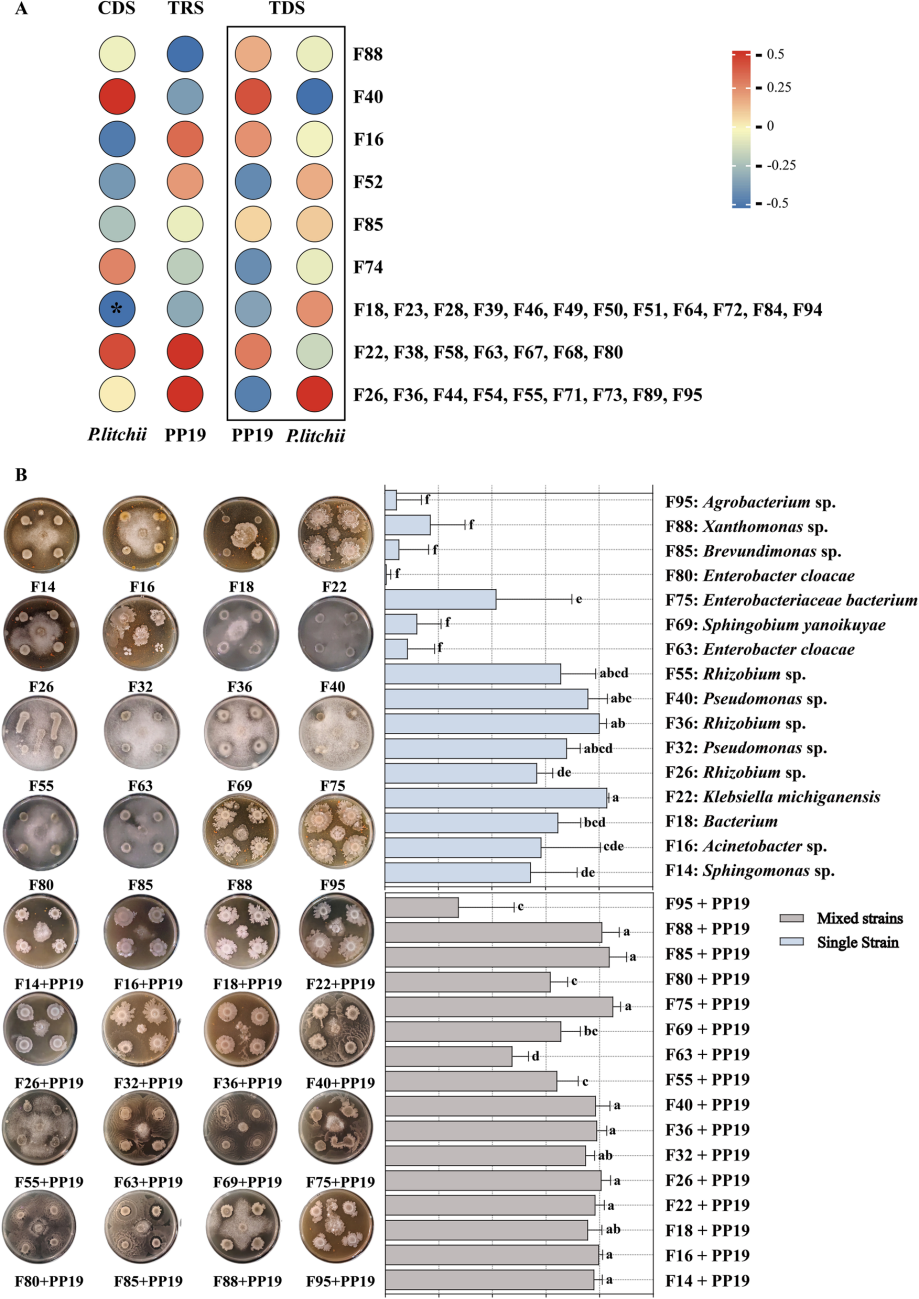
**A** Correlation analysis between 83 cultivable bacterial strains isolated from litchi peel and PP19 or SC18 under different treatments. **B** Direct antifungal activity of 16 candidate biocontrol litchi peel bacteria, as single strains and in combination with PP19, against *Peronophythora litchii*

To investigate the mechanisms by which PP19 regulates litchi exocarps bacteria to inhibit *P*. *litchii*, we analyzed interactions between differentially abundant genera and OTUs. Sixteen strains (F14, F16, F18, F22, F26, F32, F36, F40, F55, F63, F69, F75, F80, F85, F88, F95) that interacted with both PP19 and SC18 were selected as candidates from 83 litchi.

exocarp bacterial strains (Fig. 6A). We evaluated the direct antifungal ability of these 16 candidate strains against SC18 through co-culture, and assessed their biocontrol efficacy on detached litchi leaves. The inhibition by single candidate strains varied from 0.74% to 83.1%, with significant differences among strains. Notably, F22, F36, and F40 showed inhibition levels higher than PP19 (75%), with F22 achieving the highest level (83.1%). The inhibition range of mixed candidate strains, and combinations of candidate strains with PP19, varied from 27.5% to 85.2%. Compared to the control, the inhibition by mixed strains was generally higher than those of single strains, except for F22, F36, and F55, whose mixed strains showed reduced inhibition levels compared to single strains (Fig. 6B).

**Fig. 7:**
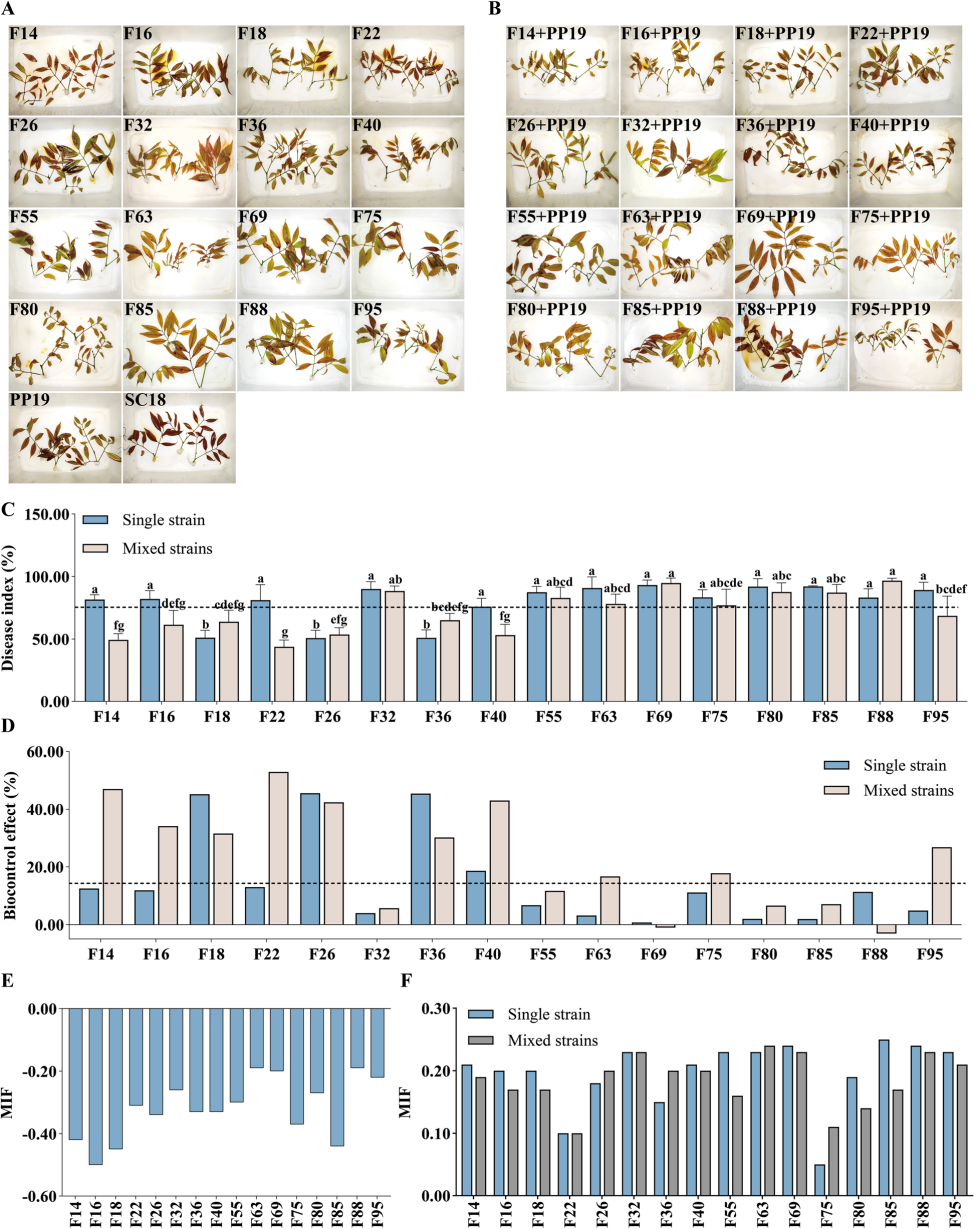
Biocontrol activity of (**A**) single strains or (**B**) mixed with PP19 against litchi downy blight on detached leaves. **C** disease incidence index and (**D**) biocontrol index for these interactions; MIF values of the 16 candidate strains as (**E**) single strains and (**F**) in mixed strains with PP19 in the presence or absence of the pathogen SC18

The biocontrol efficacy of candidate strains on detached litchi leaves showed significant changes in SC18 infection rates compared to the control after placing with the 16 candidate strains or each mixed with PP19 (Fig. 7A-C). The efficacy of single strains F22, F36, and F18 was significantly higher than PP19 (75%), with F18 achieving the highest biocontrol index (51%). Among the strains mixed with PP19, some candidates, such as F14, F16, and F22, showed significantly improved biocontrol levels compared to single strains (Fig. 7D). We also evaluated the interaction strength between candidate strains and PP19 through co-culture to determine whether PP19 directly interacted with candidate strains to influence *P*. *litchii* infection. The mean interaction factor (MIF) between candidate strains and PP19 shifted from negative (antagonistic) to positive after SC18 treatment, indicating a strong positive interaction resulting in less disease (Fig. 7E, F). These results suggested that in the presence of *P*. *litchii*, PP19 has positive interactions with the 16 candidate strains, enhancing antifungal and biocontrol capabilities to reduce *P*. *litchii* infection on litchi leaves.

### Interactions between PP19 and functional bacteria on litchi exocarps

**Fig. 8:**
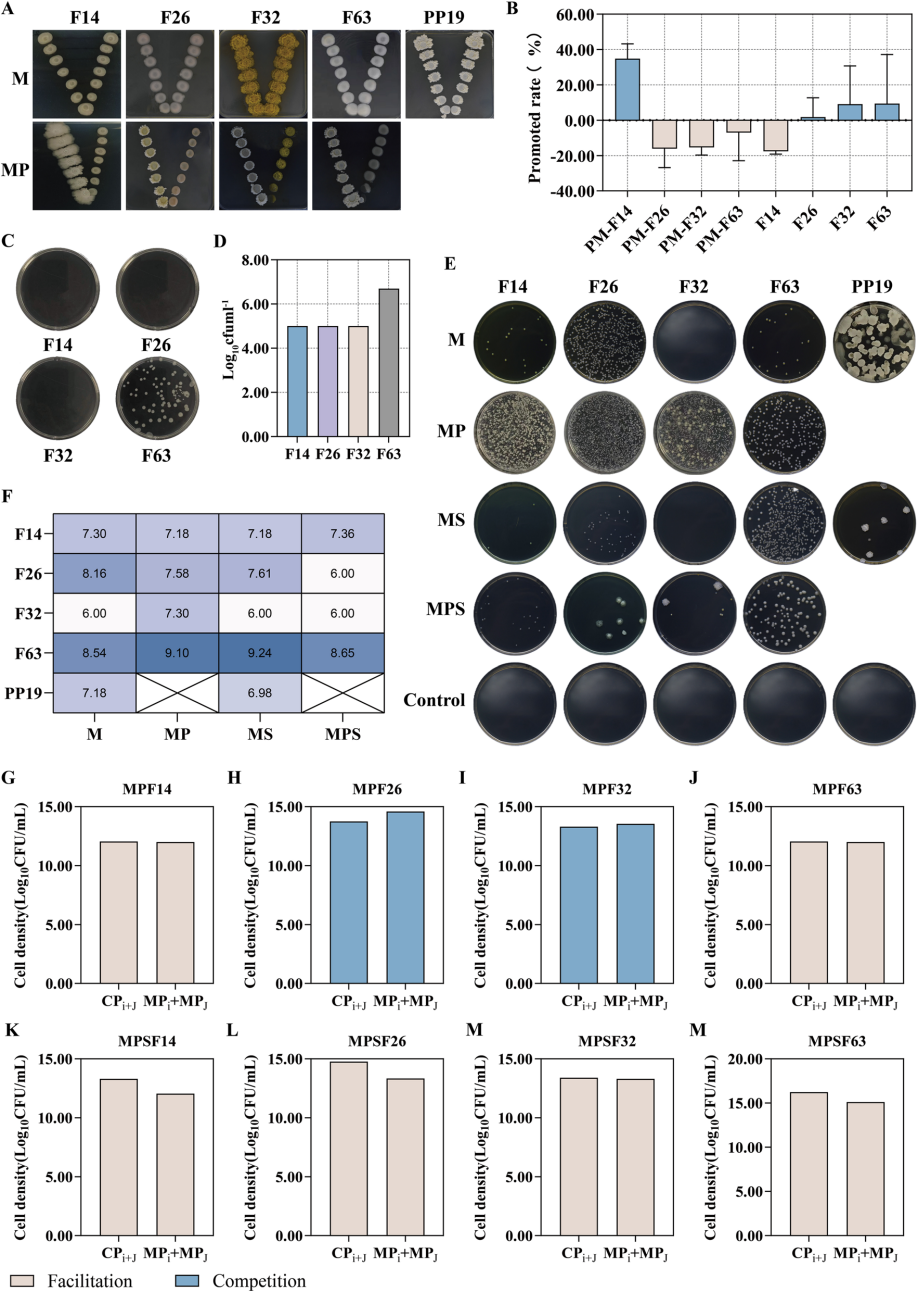
Analysis of (**A** and **B**) attraction between PP19 and the four representative strains; (**C** and **D**) the attraction exerted by PP19 supernatant toward the four representative strains; (**E** and **F**) co-culture experiments of PP19 with the four representative strains; interaction relationships between PP19 and the four representative strains analyzed through co-culture under the (**G**, **H**, **I**, **J**)presence or (**K**, **L**, **M**, **N**) absence of the pathogen

To better understand the interactions between PP19 and litchi exocarp bacteria, we conducted a comprehensive evaluation involving plate antagonism, liquid co-culture, and leaf biocontrol efficacy experiments. Four strains showed distinct correlations (F14: positively correlated with PP19 and negatively correlated with *P. litchii*; F26: negatively correlated with both PP19 and *P. litchii*; F32: negatively correlated with PP19 and positively correlated with *P. litchii*; and F63: positively correlated with both PP19 and *P. litchii*). These were selected for further study. We assessed the interactions between PP19 and these four strains by analyzing their attraction and chemotactic effects. In the attraction experiment, F14 and PP19 exhibited mutual attraction, indicating an interaction between them (Fig. 8A, B). In the chemotaxis experiment, PP19's supernatant attracted F63, suggesting that PP19's extracellular secretions have a chemotactic effect on F63 (Fig. 8C, D). These results suggested that PP19 can directly interact with F14 and F63 through possibly different non-competitive mechanisms. We also analyzed the interaction types between PP19 and the four strains under pathogen presence using liquid co-culture experiments (Fig. 8E-M). The results showed that in the absence of *P. litchii*, PP19 had a facilitative relationship with F14 and F63 and a competitive relationship with F26 and F32. However, in the presence of the pathogen, interactions between PP19 and all four strains all shifted to facilitative relationships. These findings indicated that the presence of *P. litchii* altered the interactions between PP19 and F26/F32, potentially enhancing PP19's inhibitory effect on *P. litchii*. Facilitation is used here in the sense of Li [41], refers to a facilitative interaction where MP_i_ + MP_j_ < CP_i+J_.

**Fig. 9:**
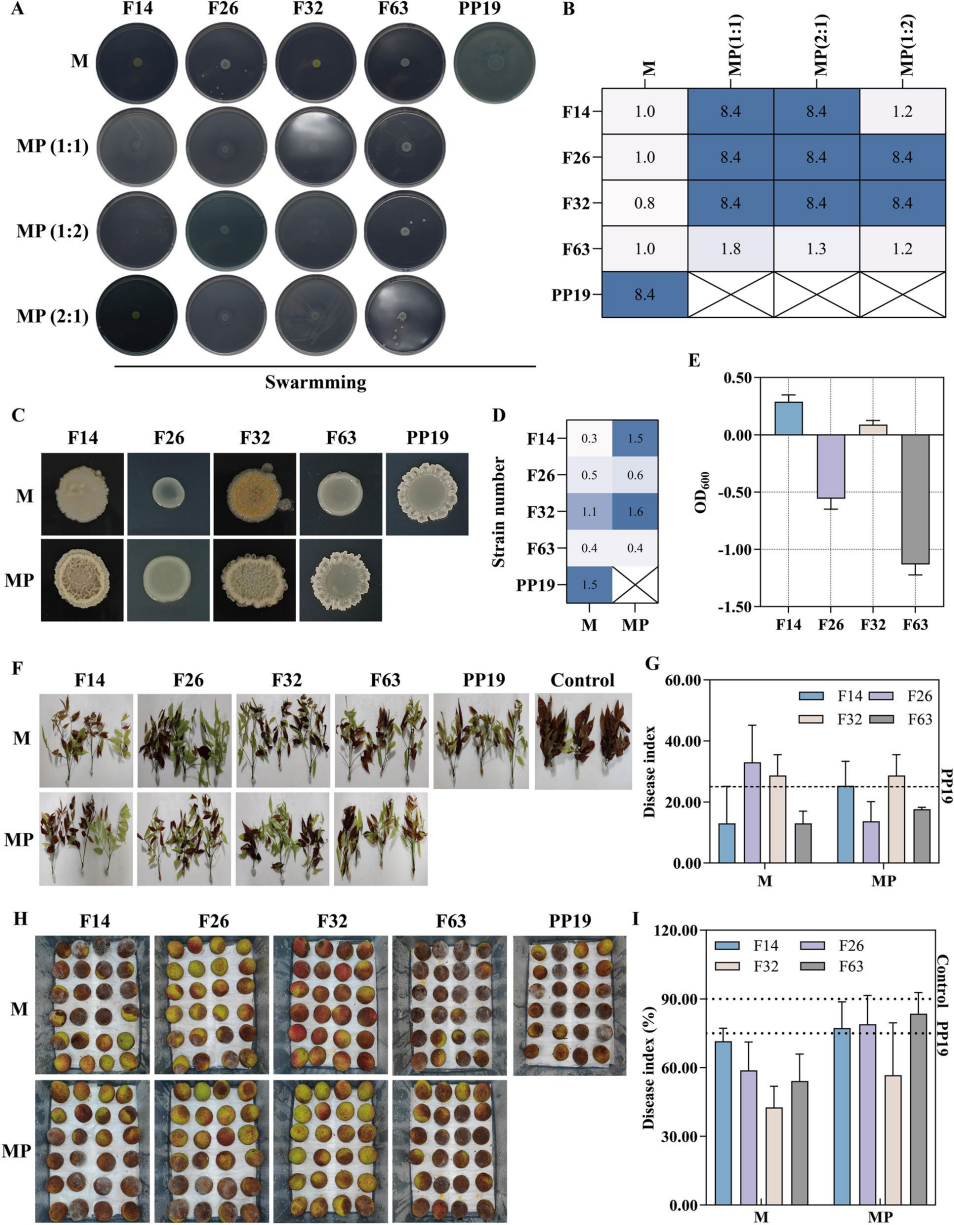
Effects of PP19 on (**A** and **B**) the swarming ability of the four representative strains and (**C**, **D**, **E**) biofilm production of the four representative strains; biocontrol efficacy against *Peronophythora litchii* on detached litchi leaves (**F**) without or (**H**) with PP19, and disease incidence index (**G**) without or (**I**) with PP19

To further understand the effect of PP19 on the physiological characteristics and behavior of litchi exocarp bacteria, we analyzed its impact on the motility and biofilm production of four strains. The results showed significant differences in swarming ability among the strains. F14 exhibited strong swarming ability under various treatments, which was further enhanced when mixed with PP19 in different proportions. Strains F26, F32, and F63 lacked intrinsic swarming ability but gained swarming motility when mixed with PP19. Among these, the swarming ability of the F63 + PP19 mixture was lower than that of the other strains (Fig. 9A, B). Biofilm production analysis revealed significant differences, with F14 producing the highest amount of biofilm. When mixed with PP19, biofilm production increased significantly in all four strains, with F14 and F26 showing the greatest increases, although still lower than PP19 alone (Fig. 9C-E). Finally, using detached litchi leaves and fruits, we evaluated the biocontrol efficacy of the four individual strains and their mixtures with PP19 against *P. litchii* by calculating disease incidence. In the detached leaf biocontrol experiment, F14 and F63 alone showed better efficacy than PP19, while mixtures of F26 and F63 with PP19 exhibited improved efficacy (Fig. 9F, G). In the fruit biocontrol experiment, all four strains alone performed better than PP19, and both single and mixed strains were effective in controlling *P. litchii*, with no significant differences observed (Fig. 9H, I). These results suggested that PP19 interacted beneficially with four representative litchi exocarps bacteria perhaps through different mechanisms, enhancing their motility and biofilm production to varying degrees.

## Discussion

Previous studies on litchi downy blight have primarily focused on pathogen identification and chemical control [42]. However, prolonged reliance on chemical control can lead to pathogen resistance and environmental pollution, underscoring the urgent need for sustainable biocontrol strategies [43–45]. Although current research on biocontrol has made some progress, the mechanisms by which biocontrol bacteria regulate plant microbial communities to suppress diseases remain poorly understood. Specifically, the influence of the litchi exocarp micro-ecological structure on the growth of *P. litchii*, and the interactions among biocontrol bacteria, pathogens, and other bacterial strains, remain largely unexplored. These issues represent critical gaps in current research on litchi downy blight.

In this study, we investigated the potential mechanisms by which *Bacillus amyloliquefaciens* PP19 can inhibit litchi downy blight. Through systematic microbiome analysis and bacterial interaction studies, we elucidated potential biocontrol mechanisms of *B. amyloliquefaciens* PP19. Microbiome analysis revealed that PP19 can regulate the core microbial community on litchi exocarps (e.g., *Actinomycetospora*, *Paenibacillus*, and *Spirosoma*), maintaining microecological stability and preventing pathogen-induced diversity imbalances. Studies reported by Situ [6] indicated that the colonization of PP19 altered the microbial community composition on litchi exocarps, which may help inhibit the infection of *P. litchii*. Moreover, significant changes in the microbiome were observed on litchi fruit surfaces at 60 h after PP19 treatment. Different from the findings of Situ, our statistical analysis using T-tests for pairwise comparisons showed that PP19 treatment at 72 h significantly induced changes in the litchi exocarp microbiome, whereas changes in the microbiome of litchi exocarps treated for 60 h were not significant (Supplemental Figure 3). We suggest that the litchi cultivar used in the study and the environmental conditions of the litchi planting site were the main causes of these differing results. Previous studies confirmed that biofilms produced by biocontrol bacteria enhance bacterial adhesion, stability, and environmental stress resistance on plant surfaces, thereby facilitating their biocontrol efficacy [46, 47]. For example, *Pseudomonas fluorescens* colonizes wheat root surfaces via biofilm formation and secretes pyoverdine to sequester environmental iron ions, thereby restricting the growth of the pathogenic fungus *Gaeumannomyces graminis* due to iron deficiency [48]. Thereby, bacterial biofilm production may play a critical role in biocontrol mechanisms. Through bacterial interaction networks and functional prediction, we found that PP19 enhanced the inhibitory effect against *P. litchii* by promoting biofilm formation and motility of specific bacteria on litchi fruit peels, particularly those with positive interactions (e.g., *italics Sphingomonas sp*. F14).Previous studies have demonstrated that *Bacillus amyloliquefaciens* can enhance biofilm formation by itself and by other beneficial microorganisms by secreting signaling molecules, extracellular polysaccharides (EPS), surface proteins, and antimicrobial peptides [49–51]. For instance, *Bacillus amyloliquefaciens* promotes biofilm formation in *Staphylococcus aureus* via secreted antimicrobial peptides and exogenous substances such as lipopeptides, surfactants, and EPS; these secretions facilitate biofilm establishment by *S. aureus* in complex environments and enhance its antibiotic resistance [52]. Additionally, *Bacillus amyloliquefaciens* produces organic acids that alter local soil pH, thereby promoting biofilm formation in *Actinobacteria* and improving their survival in soil environments [53]. In this study, we utilized 16S rRNA sequencing to predict the functional roles of PP19 in modulating bacterial communities on litchi exocarps. The promotive effects of PP19 on biofilm formation in selected bacterial strains were experimentally validated; however, the biological mechanisms by which PP19 enhances biofilm formation in other bacteria to collectively suppress *Peronophythora litchii* infection remain to be elucidated and warrant in-depth investigation. In contrast to previous studies, this work not only investigated the direct inhibitory capacity of PP19 against P*. litchii* infection in litchi fruits but also revealed a novel biocontrol mechanism based on microbial community regulation through microbiome analysis, microbial interactions, and functional profiling.

The stability of microbial communities is critical for plant health and disease control. Previous studies have shown that diverse microbial communities can suppress pathogen growth through mechanisms such as competition, antagonism, and resource occupation, thereby enhancing ecosystem stability [54, 55]. However, external factors such as pathogen infection and pesticide use can disrupt the stability of plant surface microbial communities, reducing beneficial microbes and increasing the risk of harmful microbial invasion [56]. Our findings align with previous reports, confirming that *P. litchii* infection significantly disturbs the bacterial community on litchi exocarps. This disturbance is reflected in a notable increase in bacterial diversity (Shannon and Simpson indices), suggesting that *P. litchii* infection may alter microbial community structure allowing for or associated with increased disease. Compared to previous studies, our research further revealed that the imbalance in bacterial diversity caused by *P. litchii* infection may contribute to or be associated with the development of litchi downy blight. Although diverse microbial communities can inhibit pathogen infection [22, 57], our results showed that *P. litchii* infection increased bacterial diversity. This observation could suggest that the increased diversity driven by *P. litchii* might be associated with destabilized microbial community dynamics, where the concurrent proliferation of specific genera could potentially create ecological niches favoring disease progression. These patterns align with the ecological theory that both alpha diversity and community structure collectively influence ecosystem functioning. Our findings expand on previous research regarding the relationship between pathogens and microbial diversity [58, 59].

Our study suggests a significant role of the biocontrol bacterium PP19 in maintaining the stability of the litchi exocarp microbial community. Compared to untreated litchi exocarps, the PP19-pretreated group maintained stable Shannon and Simpson indices, implying that PP19 prevented bacterial diversity imbalance caused by *P. litchii* infection by suppressing the growth of specific genera associated with the pathogen. This is in congruence with mechanisms proposed by Pieterse [60] and Hou [61], where biocontrol bacteria inhibit pathogens through antibiotic production, nutrient competition, and activation of plant immune responses. However, our study extended this understanding by revealing PP19’s crucial role in maintaining diversity through changes in the microbial community structure of litchi exocarps. Interaction network analysis further demonstrated that the interactions between PP19 and litchi exocarp bacteria changed in the presence of the pathogen. In the absence of the pathogen, PP19 exhibited negative interactions with 16 beneficial bacteria, but upon pathogen introduction, these interactions shifted to synergism. This suggested that PP19's biocontrol activity may be activated by *P. litchii* for full effectiveness. This phenomenon revealed that pathogens not only directly affect plant health but also indirectly regulate interactions between biocontrol agents and other beneficial microbes [62, 63]. This mechanistic discovery provides a new research direction for future biocontrol strategies.

This study found that PP19 treatment significantly altered the bacterial community structure on litchi exocarps. Unlike *P. litchii* treatment, PP19 reduced bacterial diversity while increasing the abundance of specific beneficial genera, particularly *Bacillus*. This aligns with the findings of Geat [64], who showed that specific biocontrol bacteria can promote the growth of beneficial bacteria through mechanisms such as symbiotic relationships and metabolite sharing. Our study systematically revealed interactions between PP19 and the litchi exocarp microbial community by integrating culture-based methods with 16S rRNA analysis, providing critical evidence for the application of biocontrol bacteria in disease management. In conclusion, building on previous studies, this research confirmed the disruptive effect of *P. litchii* on the microbial community of litchi exocarps, identified a critical role for PP19 in maintaining community stability, and uncovered a novel mechanism of interaction between pathogens and biocontrol bacteria. Compared to prior studies, this research advanced our understanding of the relationships between microbial diversity and disease control, as well as the mechanisms of biocontrol, providing theoretical support and practical guidance for the application of biocontrol in agricultural disease management.

Although this study has made progress in elucidating the relationship between the stability of the litchi exocarp microbial community and disease control, certain limitations remain and future research is needed. The microbial community on plant surfaces includes diverse microorganisms, and their interactions influence the community's composition and stability [65, 66]. However, this study primarily relied on partial 16S rRNA sequencing, which may potentially limit the characterization of non-bacterial components (e.g., fungi, viruses) and the resolution of bacterial taxa to species level—factors that could affect the interpretation of community complexity. It is also worth noting that functional predictions based on partial 16S regions (e.g., via tools like PICRUSt [67]) might face challenges in capturing species-specific metabolic traits, as genus-level taxonomic assignments may not fully reflect functional gene variations. This technical constraint could introduce uncertainties in inferring key biocontrol functions (e.g., pathogen inhibition pathways), highlighting the need for integrated multi-omics approaches in future studies. Additionally, the interaction mechanisms between PP19 and other beneficial microorganisms remain insufficiently elucidated, especially under pathogen activation, where in-depth molecular and metabolic-level validation is lacking. Future research should explore these aspects.

Plant health is closely linked to microbial diversity. Based on the findings of this study, it is crucial to further explore the impact of *P. litchii* on the microbial community of litchi exocarps and the mechanisms of the biocontrol bacterium PP19 from multiple perspectives. First, regarding the phenomenon of increased microbial diversity on litchi exocarps following *P. litchii* infection, we propose investigating the mechanisms underlying this diversity increase. It is essential to focus on the roles and functions of specific microbial groups in disease development. Second, the interactions between *P. litchii* and key beneficial microorganisms should be assessed to determine whether *P. litchii* enhances its potential pathogenicity by suppressing beneficial microbial populations. In this study, we also identified a unique interaction mechanism of *P. litchii* on PP19, suggesting that PP19 may require activation by *P. litchii* to interact with beneficial exocarp microbes. Further research is needed to confirm this interesting phenomenon.

## Supplementary Information

Supplementary Material 1. Supplementary Fig. S1. Comparative changes in the relative abundance of *Bacillus amyloliquefaciens* PP19 and *Peronophythora litchii* on litchi exocarps under different treatments.

Supplementary Material 2. Supplementary Fig. S2. BOX-PCR genomic fingerprinting of 83 bacterial isolates from litchi exocarps. DNA molecular weight marker (1-kb ladder).

Supplementary Material 3. Supplementary Fig. S3. T-test was used to analyze the significant differences in α-diversity of *litchi* exocarp samples treated with PP19 at 60 h (A-E) and 72 h (F-J) post-treatment.

Supplementary Material 4. Supplementary Table S1. Number of strains isolated from litchi microenvironments by PP19 enriched.

## Data Availability

All data included in this study are available upon request by contact with the corresponding author. 16S rRNA sequencing data can be accessed on SRA under the accession PRJNA1229107. Please visit this link to access the 16S rRNA sequencing data, https://dataview.ncbi.nlm.nih.gov/object/PRJNA1229107?reviewer=9njqjndus31jgu2e89pv02oeoi.
