# Supplementary figures and images for: Bacillus amyloliquefaciens PP19 regulation of microbial communities and suppression of Peronophythora litchii
Source: Microbiome. 2025 Dec 18;14:40. doi: 10.1186/s40168-025-02239-y (PMC12829244; doi:10.1186/s40168-025-02239-y)

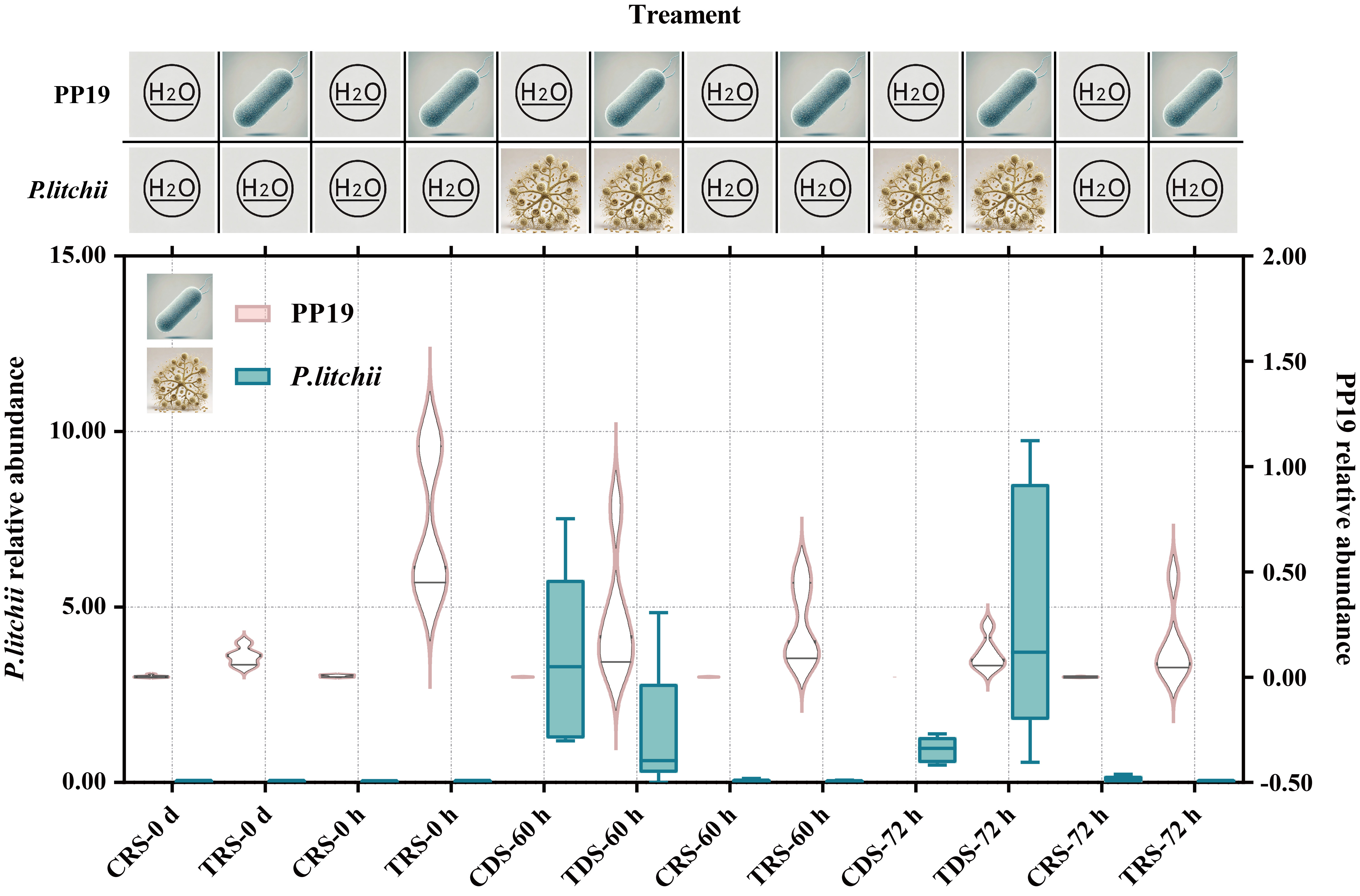

Supplement: Supplementary file 2 — Supplementary Material 1. Supplementary Fig. S1. Comparative changes in the relative abundance of Bacillus amyloliquefaciens PP19 and Peronophythora litchii on litchi exocarps under different treatments. [file 40168_2025_2239_MOESM1_ESM.jpg]

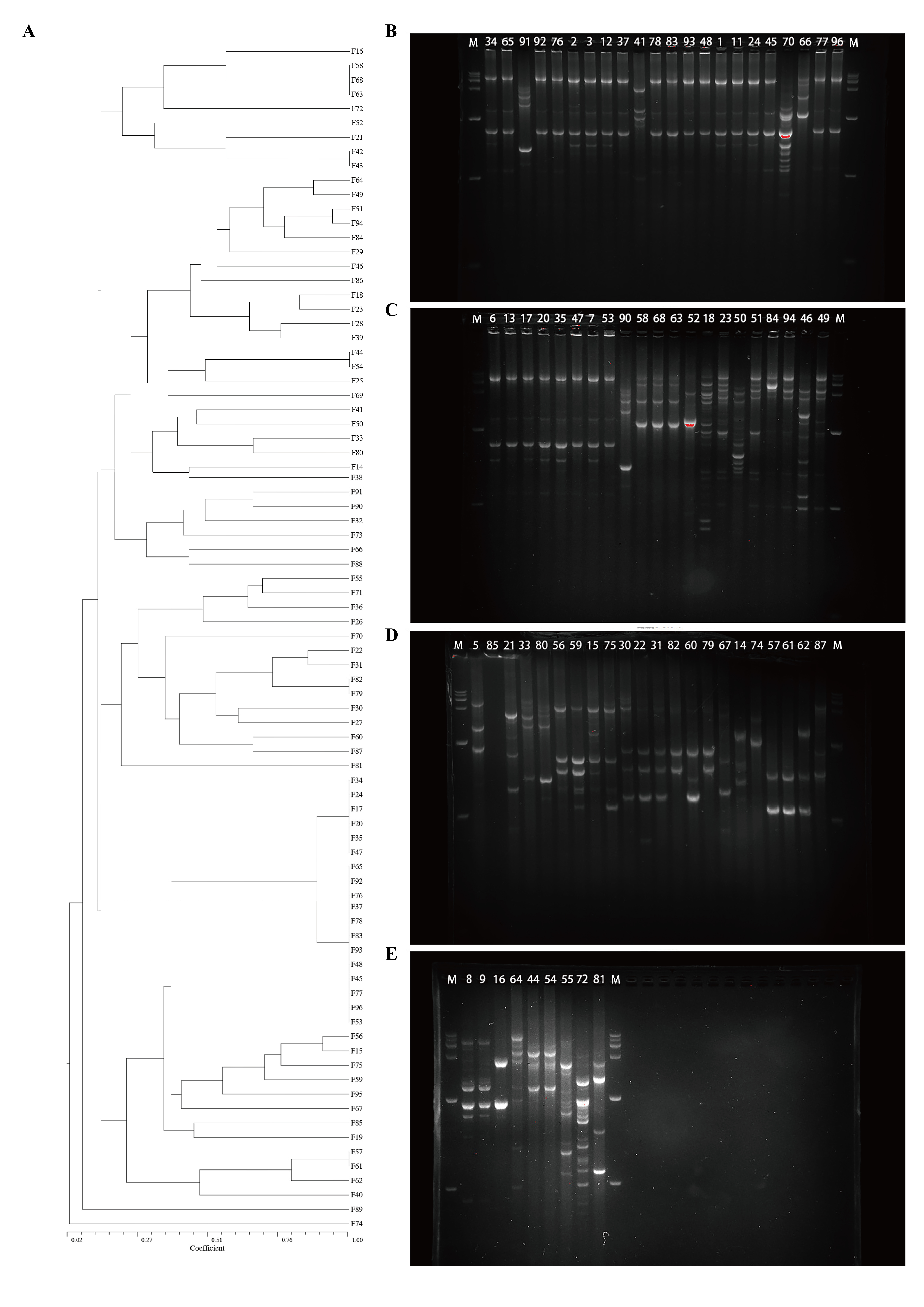

Supplement: Supplementary file 3 — Supplementary Material 2. Supplementary Fig. S2. BOX-PCR genomic fingerprinting of 83 bacterial isolates from litchi exocarps. DNA molecular weight marker (1-kb ladder). [file 40168_2025_2239_MOESM2_ESM.jpg]

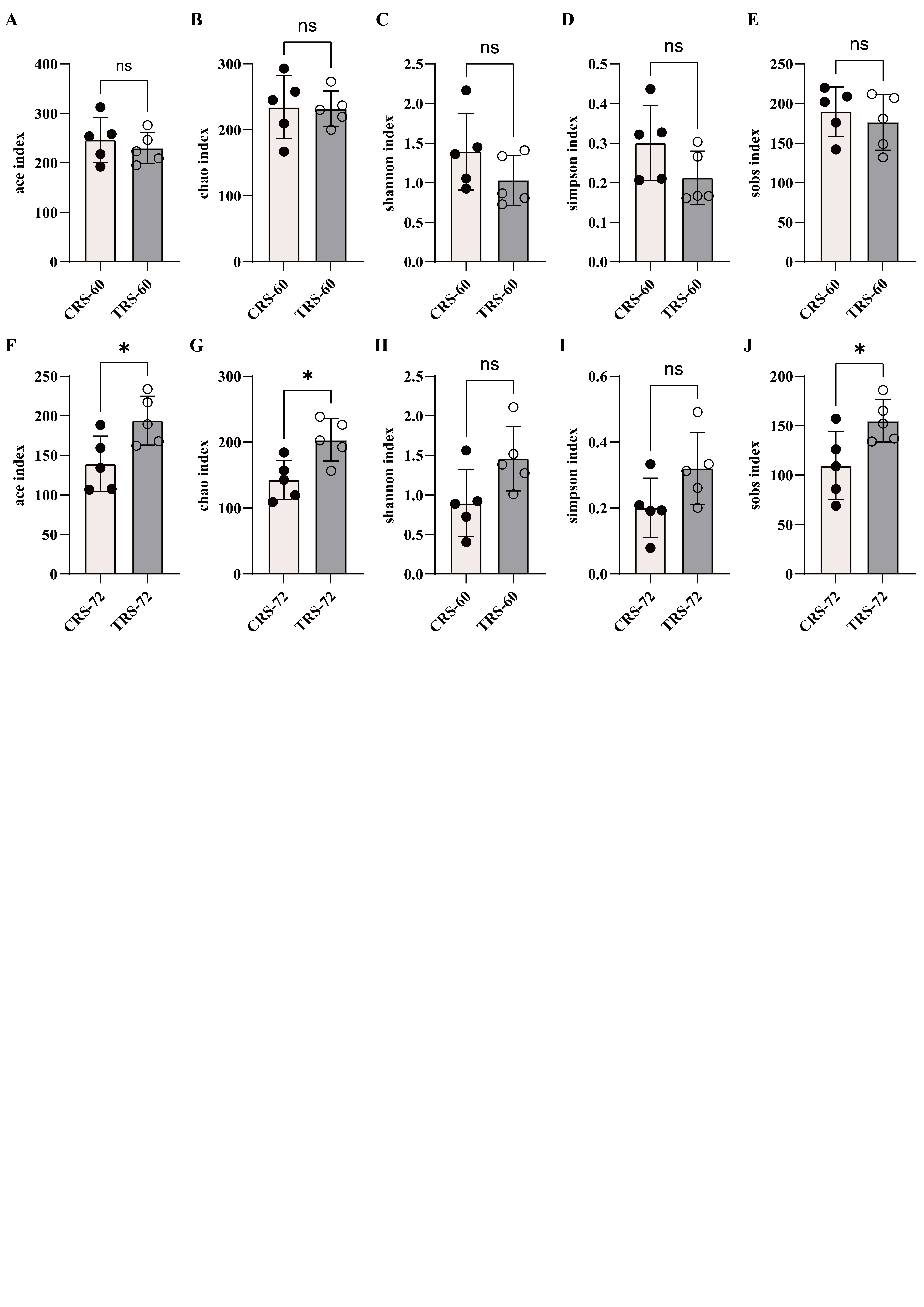

Supplement: Supplementary file 4 — Supplementary Material 3. Supplementary Fig. S3. T-test was used to analyze the significant differences in α-diversity of litchi exocarp samples treated with PP19 at 60 h (A-E) and 72 h (F-J) post-treatment. [file 40168_2025_2239_MOESM3_ESM.jpg]
