# Supplementary material for: Bacillus amyloliquefaciens PP19 regulation of microbial communities and suppression of Peronophythora litchii
Source: Microbiome. 2025 Dec 18;14:40. doi: 10.1186/s40168-025-02239-y (PMC12829244; doi:10.1186/s40168-025-02239-y)
Supplement: Supplementary file 5 — Supplementary Material 4. Supplementary Table S1. Number of strains isolated from litchi microenvironments by PP19 enriched. [file 40168_2025_2239_MOESM4_ESM.docx]

**Supplementary Table S1**. Number of strains isolated from litchi microenvironments by PP19 enriched

| **Habitat** | **NIS** | **Strain No.** | **Bacterial Population Density (105 CFU/g)** | **Percentage (%)** | **NIS** | **Strain No.** | **Bacterial Population Density (105 CFU/g)** | **Percentage (%)** | **NIS** | **Strain No.** | **Bacterial Population Density (105 CFU/g)** | **Percentage (%)** | **NIS** | **Strain No.** | **Bacterial Population Density (105 CFU/g)** | **Percentage (%)** |
| --- | --- | --- | --- | --- | --- | --- | --- | --- | --- | --- | --- | --- | --- | --- | --- | --- |
| **60 hpi** | 6 | F14-F19 | 17.7 | 31.6 | 9 | F56-F64 | 83.3 | 42.9 | 6 | F34-F39 | 9.7 | 26.1 | 5 | F78-F82 | 38.0 | 25 |
| **72 hpi** | 7 | F20-F26 | 23.7 | 36.8 | 5 | F65-F69 | 40.0 | 23.8 | 8 | F40-F47 | 16.0 | 34.8 | 6 | F83-F88 | 7.7 | 30 |
| **84 hpi** | 4 | F27-F30 | 195.0 | 21.1 | 5 | F70-F74 | 71.0 | 23.8 | 5 | F48-F52 | 15.7 | 21.7 | 6 | F89-F84 | 6.3 | 30 |
| **0 hpi** | 2 | F31-F32 | 4.7 | 10.5 | 2 | F95-F96 | 3.0 | 9.5 |  |  |  |  |  |  |  |  |
| **0 hpt** |  |  |  |  |  |  |  |  | 4 | F33\F53-55 | 8.0 | 17.4 | 3 | F75-F77 | 2.7 | 15 |
| **Total number** | 19 |  |  |  | 21 |  |  |  | 23 |  |  |  | 20 |  |  |  |
| **Treatments** | **PP19+SC18（TDS)** | | | | **CK+SC18(CDS)** | | | | **PP19(TRS)** | | | | **CK(CRS)** | | | |

Note: hpt (hour post treatment): time after inoculation with the strain; hpi (hour post inoculation): time after treatment; DS (Diseased sample): fruit inoculated with *P. litchii*; RS (Remain fresh sample): fruit not inoculated with *P. litchii*.; TDS: habitat of bacteria enriched by PP19 pretreatment after inoculation with SC18; CDS: habitat of bacteria enriched by the control but after inoculation with SC18; TRS: habitat of bacteria enriched by PP19 pretreatment; CRS: habitat of bacteria enriched by the control; NIS: Number of Isolated Strains.
